# Psychoedukativer schematherapeutischer Ansatz bei Psoriasis und atopischer Dermatitis: Eine randomisierte kontrollierte Pilot‐Crossover‐Studie

**DOI:** 10.1111/ddg.70020x

**Published:** 2026-10-05

**Authors:** Regina von Spreckelsen, Hila Emmert, Jan Hartmann, Matthias Hübenthal, Stephan Weidinger, Ulrich Mrowietz, Eva M. J. Peters, Sascha Gerdes

**Affiliations:** ^1^ Zentrum für entzündliche Hauterkrankungen, Klinik für Dermatologie Venerologie und Allergologie, Universitätsklinikum Schleswig‐Holstein Campus Kiel Kiel Deutschland; ^2^ Labor für Psychoneuroimmunologie, Klinik für Psychosomatik und Psychotherapie, Universitätsklinikum Gießen Gießen Deutschland; ^3^ Zentrum für Innere Medizin und Dermatologie, Klinik für Psychosomatische Medizin und Psychotherapie, Charité – Universitätsmedizin Berlin Freie Universität Berlin und Humboldt‐Universität zu Berlin Berlin Deutschland

**Keywords:** atopische Dermatitis, depressive Symptome, integrierte Versorgung, Kurzzeittherapie, Lebensqualität, Pilotstudie, Psoriasis, Psychodermatologie, Psychoedukation, Schematherapie, atopic dermatitis, brief psychotherapy, depressive symptoms, integrated care, pilot trial, psoriasis, psychodermatology, psychoeducation, quality of life, schema therapy

## Abstract

**Hintergrund und Ziele:**

Atopische Dermatitis und Psoriasis sind chronisch‐entzündliche Hauterkrankungen, die häufig mit psychischem Stress einhergehen. Integrative Versorgungsmodelle fehlen weitestgehend. Diese randomisierte Pilotstudie hatte zum Ziel, zu der dermatologischen Behandlung eine begleitende schematherapeutisch ausgerichtete psychoedukative Kurzzeitintervention für dermatologische Patienten zu entwickeln und zu testen.

**Patienten und Methodik:**

Patienten aus einem ambulanten Entzündungszentrum mit einem PHQ‐2 (Patient Health Questionnaire‐2) Score ≥ 3 wurden randomisiert und einer Interventions‐ oder Kontrollgruppe zugewiesen. Die Interventionsgruppe erhielt drei standardisierte Sitzungen mit den Schwerpunkten maladaptive Schemata, Bewältigungsstrategien, Psychoedukation und emotionsfokussierte Techniken (zum Beispiel Stuhldialoge, Imagination).

**Ergebnisse:**

19 Betroffene erhielten die Intervention, 13 gehörten zur Kontrollgruppe. Nach der Intervention wurden signifikante Verbesserungen der dermatologischen Lebensqualität (DLQI), des subjektiven Wohlbefindens (WHO‐5) und der depressiven Symptome (PHQ‐9, BDI‐II) beobachtet. Sie waren weitgehend unabhängig vom Schweregrad der Erkrankung (PASI, EASI). Qualitative Rückmeldungen hoben die Benutzerfreundlichkeit, das Erlernen spezifischer Techniken und eine vertrauensvolle therapeutische Beziehung hervor.

**Schlussfolgerungen:**

Eine kurze psychologische Intervention reduziert die psychische Belastung bei dermatologischen ambulanten Patienten signifikant. Weitere Studien sollten die langfristigen Auswirkungen und eine breitere Umsetzung untersuchen.

## EINLEITUNG

Chronische entzündliche Hauterkrankungen gehen häufig mit psychischen Erkrankungen wie Depressionen, Angststörungen oder somatischen Stressstörungen einher.^1^, ^2^, ^3^, ^4^ Im Durchschnitt weist etwa ein Drittel aller Patienten mit Hauterkrankungen psychische Symptome auf. Die Lebensqualität ist noch häufiger beeinträchtigt und wird auch in pharmakologischen Studien als therapeutisches Ergebnis berücksichtigt.^5^ Eine verminderte gesundheitsbezogene Lebensqualität (HRQoL) korreliert jedoch nicht unbedingt mit dem Schweregrad der Erkrankung, was ebenfalls dazu beitragen kann, dass die psychische Belastung der Patienten im somatischen Kontext oft nicht die angemessene Aufmerksamkeit erhält.^6^ Anstatt Unterstützung bei der Bewältigung ihrer Krankheit zu erhalten, fühlen sich Patienten oft stigmatisiert. Der daraus resultierende psychische Stress spielt eine wichtige Rolle.^7^ Eine schlechte psychische Gesundheit kann auch zu einem sich selbst verstärkenden Kreislauf führen, in dem Hautsymptome durch Stress verschlimmert werden und eine verminderte gesundheitsbezogene Lebensqualität zu einer verminderten Therapietreue führen kann.^8^

Stress und psychische Belastungen sind wichtige Faktoren für Patienten, und es gibt zunehmend Hinweise darauf, dass nicht nur Hautkrankheiten psychische Erkrankungen begünstigen, sondern dass psychischer Stress auch Einfluss auf die somatischen Merkmale der Erkrankung hat, wie beispielsweise Juckreiz.^9^, ^10^, ^11^, ^12^, ^13^

Psychologische Faktoren sollten bei der Diagnose und Behandlung dermatologischer Patienten berücksichtigt werden und ein integraler Bestandteil sein.^14^ Dieses Wissen spiegelt sich in der zunehmenden Forschung zu psychotherapeutischen Interventionen wider.^15^ Neue Erkenntnisse aus der Psychoneuroimmunologie liefern auch biopsychosoziale Konzepte, die die gegenseitige Verbindung zwischen Bereichen betonen, die zuvor getrennt betrachtet wurden, nämlich Hautkrankheiten einerseits und psychische Erkrankungen andererseits.^16^ Sie unterstützen den modernen Ansatz der Psychodermatologie, der davon ausgeht, dass durch den Einsatz kurzer Fragebögen Patienten, die unter Stress leiden, mit geringem Aufwand frühzeitig in ihrem Alltag identifiziert und umgehend geeigneten Behandlungsmöglichkeiten zugewiesen werden können.^17^, ^18^ Da psychotherapeutische Leistungen in der Regel zeitaufwendig sind und die Suche nach einem geeigneten Angebot viel Zeit in Anspruch nehmen kann, sind innovative Ansätze erforderlich, die mit kurzen Interventionen erste Impulse im ambulanten Bereich setzen können.

Die kognitive Verhaltenstherapie (KVT) ist in diesem Zusammenhang eine häufig untersuchte Therapiemethode.^19^, ^20^ Die Schematherapie (ST), eine Weiterentwicklung der Verhaltenstherapie von Jeffrey Young,^21^ integriert kognitive, emotionale und verhaltensbezogene Interventionen sowie emotions‐ und verhaltensbasierte Ansätze. Ein zentrales Element der Schematherapie ist der gezielte Aufbau einer therapeutischen Beziehung zwischen Therapeuten und Patient. Die Symptome, Verhaltensmuster und zentralen biografischen Ereignisse werden mithilfe der Fallkonzeption auf dem Flipchart so dargestellt, dass sie für den Patienten leicht zugänglich sind.^22^ Sie befasst sich mit frühen maladaptiven Mustern, die aus unerfüllten emotionalen Grundbedürfnissen resultieren können. Jeffrey Youngs Grundbedürfniskonzept umfasst vier Grundbedürfnisse: Bindung, Autonomie und Selbstwertgefühl sowie Spiel und Spontaneit.^21^ Gemeinsam mit dem Patienten wird ein individuelles Fallkonzept entwickelt, um dysfunktionales Bewältigungsverhalten (zum Beispiel Vermeidungsverhalten aufgrund von Hauterkrankungen) zu verstehen und zu verändern.^23^, ^24^ In einer früheren Studie konnten wir zeigen, dass eine psychotherapeutische Intervention, die auf schematherapeutischen Konstrukten basiert, die Lebensqualität der Patienten erhöht.^25^ Da psychotherapeutische Interventionen in der täglichen ambulanten dermatologischen Versorgung nicht regelmäßig angeboten werden oder für psychisch belastete Patienten ohne manifeste Depression schwer zugänglich sind, haben wir eine kurze, manualisierte, psychoedukative Intervention auf Basis der schematherapeutischen Fallkonzeption entwickelt und implementiert. Die schematherapeutische Kurzzeitintervention umfasst drei manual‐ und protokollbasierte Sitzungen, die *Ultra‐Intensive Short‐Term Schema Therapy* (ULTRA‐ISTST).

Die vorliegende Studie wurde als Pilotstudie konzipiert, um die Durchführbarkeit, Akzeptanz und vorläufigen klinischen Wirkungen der Ultra‐ISTST im ambulanten dermatologischen Kontext zu bewerten. Als Pilotstudie zielte sie darauf ab, wichtige Daten zu Rekrutierung, Screening, Therapietreue und Effektgrößenabschätzungen zu generieren, um die Konzeption und Durchführung einer zukünftigen definitiven randomisierten kontrollierten Studie zu unterstützen. Die Wahl eines Crossover‐Designs ermöglichte es allen Teilnehmern, einschließlich derjenigen, die ursprünglich der Kontrollgruppe zugewiesen worden waren, von der Intervention zu profitieren, und ermöglichte gleichzeitig Vergleiche zwischen den Gruppen innerhalb der Studie. Spezifische Ziele waren die Bewertung der Veränderungen der depressiven Symptome, der Lebensqualität und des Wohlbefindens nach der Ultra‐ISTST, die Untersuchung, ob diese Effekte unabhängig vom Schweregrad der Hauterkrankung waren, und die Bewertung des von den Patienten wahrgenommenen Nutzens der Intervention.

## PATIENTEN UND METHODIK

### Studiendesign

Diese Untersuchung wurde als randomisierte, kontrollierte Pilotstudie mit Crossover‐Design an einem einzigen Zentrum durchgeführt. Die Studie hatte einen explorativen Rahmen und zielte darauf ab, die Durchführbarkeit und vorläufigen Auswirkungen einer kurzen psychologisch‐edukativen Intervention (Ultra‐ISTST) bei erwachsenen Patienten mit Psoriasis vulgaris (PsV) oder atopischer Dermatitis (AD) zu bewerten, die erhöhte depressive Symptome aufwiesen. Die Teilnehmer wurden im Verhältnis 1:1 den Studienarmen zugewiesen. Patientenverbände oder Mitglieder vom öffentlichen Gesundheitswesen waren nicht an der Konzeption, Durchführung, Berichterstattung oder Verbreitung dieser Studie beteiligt.

Die Rekrutierung und Durchführung der Studie erfolgte zwischen dem 14. Juni 2021 und dem 14. August 2021 am Zentrum für entzündliche Hauterkrankungen des Universitätsklinikums Schleswig‐Holstein (UKSH), Campus Kiel, Deutschland. Die Studie endete wie geplant nach Abschluss der Rekrutierung und Nachbeobachtung; es kam zu keiner vorzeitigen Beendigung. Teilnahmeberechtigt waren erwachsene Patienten mit einer bestätigten Diagnose von PsV oder AD, die mindestens 3 Monate vor Studieneintritt von einem Dermatologen gestellt worden war. Das Screening auf potenzielle Teilnahmeberechtigung wurde konsekutiv während der routinemäßigen ambulanten Registrierung unter Verwendung des Patient Health Questionnaire‐2 durchgeführt (PHQ‐2).^26^

Folgende Einschlusskriterien wurden definiert: ein Alter ≥ 18 Jahre, ein PHQ‐2‐Wert von drei Punkten oder höher und die Vorlage einer schriftlichen Einverständniserklärung. Ausschlusskriterien waren das Vorliegen einer manifesten psychischen Erkrankung (zum Beispiel schwere depressive Störung, psychotische Störungen), unzureichende Deutschkenntnisse, Schwangerschaft oder Stillzeit, akute Suizidgedanken oder aktueller Drogen‐ oder Alkoholmissbrauch. Potenzielle Teilnehmer, die die Zulassungskriterien erfüllten, erhielten detaillierte Informationen zur Studie und gaben vor der Randomisierung ihre schriftliche Einverständniserklärung ab.

### Intervention

Die Teilnehmer, die randomisiert der Interventionsgruppe zugewiesen wurden, erhielten zusätzlich zur leitlinienbasierten dermatologischen Versorgung drei Einzelsitzungen mit der manualisierten, protokollbasierten Therapie (Ultra‐ISTST), die von ausgebildeten Psychotherapeuten im Abstand von 2 Wochen durchgeführt wurde. Jede Sitzung dauerte etwa 50 Minuten und umfasste Elemente aus der Schematherapie, der kognitiven Verhaltenstherapie und emotionsfokussierten Ansätzen, darunter Techniken wie Stuhldialoge und Imagination.^15^ Ein zentraler Bestandteil der Intervention war die gemeinsame Entwicklung einer individualisierten Fallkonzeption mit dem Teilnehmer (Tabelle 1).

**TABELLE 1:** Übersicht der 3 manualisierten Sitzungen.

Sitzung	Thema	Inhalt
*1*	***Beziehungsaufbau***	
	Vorstellung Ultra ISTST Intervention	Begrüßung, Einführung
	Einführung der individuellen Fallkonzeption	Konkrete Beschreibung der belastenden Lebenssituation im dermatologischen Kontext
		Fokusarbeit am Flipchart: erwachsener Modus; Wissen und Erkennen eigener Ressourcen und funktionalem Verhalten im Alltag
		Übung a: Imagination: Begegnung mit dem erwachsenen und kindlichen Selbst
		Übung für zuhause:
		Positive Alltagssituationen wahrnehmen und dokumentieren, Fallkonzept im Alltag überprüfen
*2*	***Individuelle Fallkonzeption im Kontext der dermatologischen Erkrankung***	
	Fokus Grundbedürfnisse	Fokusarbeit am Flipchart: Kindmodus: Wissen und Erkennen allgemeiner kindlicher Kernbedürfnisse im Vergleich zu den eigenen glücklichen, aber auch vulnerablen Kindanteilen
	Fokus dysfunktionale Bewältigungsstrategien	Fokusarbeit am Flipchart: dysfunktionale Bewältigungsstrategien: Wissen und Erkennen eigener Bewältigungsmuster
		Übung b: Pro/Contra‐Listen erstellen hinsichtlich eigener Bewältigungsstrategien
		Übung für zuhause a: Genussübung
		Übung für zuhause b: Selbstbeobachtung und Erkennen individueller Bewältigungsstrategien im Alltagskontext
*3*	***Individuelle Fallkonzeption***	
	Fokus „der eigene innere Kritiker“ (Elternmodus)	Fokusarbeit am Flipchart: "der eigene innere Kritiker" (Elternmodus); Erkennen von Selbstabwertung und Selbstbestrafung und Einordnung im biografischen wie aktuellen Kontext
		Übung c: Anleitung zur Selbstdurchführung eines Stuhl‐ Dialogs gegen Selbstabwertung und Selbstbestrafung
	Abschluss, Verabschiedung	Zusammenfassung wichtiger Schlüsselpunkte der individuellen Fallkonzeption und Transfer im Alltag

Die Teilnehmer der Kontrollgruppe erhielten die übliche Behandlung (TAU), die ausschließlich aus einer leitlinienbasierten dermatologischen Versorgung ohne psychotherapeutische Intervention bestand. In beiden Gruppen wurden die Entscheidungen zur dermatologischen Behandlung unabhängig von der Studienteilnahme gemäß den aktuellen klinischen Leitlinien getroffen und konnten von einer topischen Therapie bis hin zu einer systemischen biologischen Therapie reichen. Die im Rahmen der Studie eingesetzten psychometrischen Fragebögen (zum Beispiel DLQI, WHO‐5, PHQ‐9, BDI‐II) gehörten nicht zur Routineversorgung und wurden daher dem Studieninterventionspaket zugeordnet.

Nach Abschluss der Kontrollphase wurde den Teilnehmern der Kontrollgruppe die Möglichkeit geboten, unter identischen Bedingungen gemäß dem Crossover‐Design an der ULTRA‐ISTST‐Intervention teilzunehmen.

### Endpunkte

Der primäre Endpunkt war die Veränderung des PHQ‐2‐Scores vom Ausgangswert (V1) bis nach der Intervention (V4).

Sekundäre Endpunkte waren der Dermatology Life Quality Index (DLQI)^27^, der WHO‐5 Well‐Being Index^28^, das Beck Depression Inventar II (BDI‐II)^29^, und der Patient Health Questionnaire‐9 (PHQ‐9). Der Schweregrad der Erkrankung wurde bei PsV‐Patienten anhand des Psoriasis Area and Severity Index (PASI)^30^ und bei AD‐Patienten anhand des Eczema Area and Severity Index (EASI) und des SCORing Atopic Dermatitis (SCORAD) bewertet.^31^, ^32^

Die Ergebnisbewertungen wurden zu Beginn (V1) und nach Abschluss der Intervention (V4) durchgeführt. Für die Interventionsgruppe wurde 3 Monate nach Abschluss der Intervention eine zusätzliche Nachuntersuchung durchgeführt (V5). Alle Messinstrumente wurden in ihren validierten deutschen Versionen angewendet, und die Bewertungsverfahren, Aggregationsmethoden, Bereiche und klinischen Grenzwerte entsprachen den jeweiligen Originalveröffentlichungen.

### Randomisierung und verdeckte Zuteilung

Die Randomisierung erfolgte anhand eines einfachen Randomisierungsverfahrens ohne Stratifizierung oder Blockbildung. Für jeden verfügbaren Studienplatz wurde ein undurchsichtiger, versiegelter, identischer Umschlag mit der Gruppenzuweisung („Intervention“ oder „Kontrolle“) vorbereitet. Alle Umschläge wurden in einen undurchsichtigen Behälter gelegt und gründlich gemischt. Unmittelbar nachdem ein Teilnehmer seine Einverständniserklärung abgegeben hatte, wurde ein Umschlag von einem unabhängigen Mitglied des Studienpersonals gezogen, das nicht an der Rekrutierung, der Durchführung der Intervention oder der statistischen Analyse beteiligt war. Dieses Verfahren gewährleistete eine vollständige Geheimhaltung der Zuordnung bis zum Zeitpunkt der Zuweisung. Da es sich um eine Pilotstudie handelte, wurde die Stichprobengröße pragmatisch auf der Grundlage des verfügbaren Personals, der Zeit und der Therapiekapazität festgelegt. Es wurde keine formale Berechnung der Stichprobengröße durchgeführt, da das primäre Ziel darin bestand, die Durchführbarkeit zu bewerten und vorläufige Schätzungen der Effektgröße zu erstellen, um die Gestaltung einer zukünftigen definitiven Studie zu informieren. Es wurden keine Zwischenanalysen oder Abbruchrichtlinien geplant oder durchgeführt.

### Verblindung

Aufgrund der Art der Intervention konnten weder die Teilnehmer noch die behandelnden Ärzte hinsichtlich der Gruppenzuteilung verblindet werden. Die statistischen Analysen wurden jedoch von unabhängigen Statistikern durchgeführt, denen die Identität der einzelnen Patienten nicht bekannt war und die ausschließlich mit anonymisierten Gruppencodes arbeiteten.

### Statistische Auswertung

Für alle Variablen wurden deskriptive Statistiken berechnet. Gruppenvergleiche der kontinuierlichen Ergebnisse wurden mit dem Wilcoxon‐Rangsummentest durchgeführt. Werte außerhalb des 1,5‐fachen Interquartilsabstands wurden als Ausreißer betrachtet und aus der Analyse ausgeschlossen. Zusätzlich wurden lineare gemischte Effektmodelle mit zufälligen Schnittpunkten für Patienten angepasst (Modellformel: Punktzahl ∼ Besuch*Gruppe + (1|Patient)) und mit Nullmodellen (Punktzahl ∼ (1|Patient)) unter Verwendung einer Varianzanalyse (ANOVA) verglichen. Es wurden Effektgrößen und 95%‐Konfidenzintervalle berechnet. Die Analysen wurden mit R Version 4.3.1 durchgeführt, wobei gemischte Modelle mit dem lme4‐Paket angepasst wurden.^33^, ^34^ Alle statistischen Tests waren zweiseitig, und p‐Werte unter 0,05 wurden als statistisch signifikant angesehen.

Für die wichtigsten gruppenübergreifenden Analysen wurden alle Teilnehmer, die während des Studienzeitraums die Ultra‐ISTST‐Intervention erhielten, gemeinsam als Interventionsgruppe analysiert (n = 19). Dazu gehörten elf Teilnehmer, die ursprünglich in die Interventionsgruppe randomisiert worden waren, und acht Teilnehmer aus der Kontrollgruppe, die die Intervention während der Crossover‐Phase erhielten. Diese wurden mit den Teilnehmern der Kontrollgruppe (n = 13) verglichen, die während des Studienzeitraums keine Intervention erhielten. Die Analysen wurden nur für vollständige Fälle durchgeführt. Es wurden keine Subgruppen‐ oder Sensitivitätsanalysen vorab festgelegt oder durchgeführt.

Dieser Ansatz wurde bewusst gewählt, um die Anzahl der Teilnehmer in der Interventionsgruppe zu maximieren und damit die Genauigkeit der Effektgrößenabschätzungen für diese Pilotstudie zu erhöhen. Wir sind uns bewusst, dass diese Analyse von einem rein randomisierten Vergleich abweicht und zu Verzerrungen führen kann. Daher sollten die Ergebnisse mit Vorsicht interpretiert werden und nicht als endgültiger Beweis für die Wirksamkeit angesehen werden.

Das vollständige Studienprotokoll und die manualisierten Interventionsmaterialien sind auf begründete Anfrage beim korrespondierenden Autor erhältlich (Tabelle 1).

### Risikobewertung

Es wurde keine formelle Überwachung unerwünschter Ereignisse durchgeführt, da es sich um eine nichtpharmakologische Intervention handelte, die als risikoarm eingestuft wurde. Bei jedem Studienbesuch beurteilte der behandelnde Dermatologe den allgemeinen Gesundheitszustand des Teilnehmers. Es wurden keine interventionsbedingten unerwünschten Ereignisse gemeldet.

### Ethikvotum

Die Studie wurde von der Ethikkommission der Medizinischen Fakultät der Universität Kiel genehmigt (Referenznummer D 473/21). Die Studie wurde nicht in einem öffentlichen Studienregister registriert, da sie kein Arzneimittel oder Medizinprodukt betraf und gemäß den geltenden lokalen Vorschriften nicht registrierungspflichtig war.

## ERGEBNISSE

Die Untersuchung ergab, dass insgesamt 141 von 885 Patienten einen PHQ‐2‐Wert von ≥ 3 Punkten aufwiesen. Von diesen positiv getesteten Patienten hatten 26 (18,5%) eine bekannte Depression und kamen aufgrund der Einschlusskriterien nicht für unsere Studie in Frage. Von den übrigen positiv getesteten Patienten waren 79 (56,0%) offen für eine Intervention, während 36 (25,5%) kein Interesse daran hatten. Von den 24 verfügbaren Ultra‐ISTST‐Behandlungsplätzen wurden elf Patienten randomisiert der Interventionsgruppe und 13 der Kontrollgruppe zugewiesen (Abbildung 1). Die übrigen positiv getesteten Patienten wurden über die Ergebnisse ihres Screening‐Fragebogens informiert und gegebenenfalls aufgefordert, sich einer weiteren Untersuchung durch ihren Hausarzt oder einen Psychologen zu unterziehen. Nach Abschluss der Intervention nahmen weitere 8 Personen aus der Kontrollgruppe das Interventionsangebot an, sodass insgesamt 19 Patienten die Intervention erhielten. Alle unten aufgeführten Vergleiche zwischen den Gruppen basieren auf diesen beiden Gruppen (Intervention n = 19; Kontrolle n = 13). Die Merkmale der Stichprobe aller eingeschlossenen Patienten sind in Tabelle 2 dargestellt. Während des Studienzeitraums gab es bei keinem Patienten Änderungen der dermatologischen Therapie, einschließlich systemischer und topischer Behandlungen.

**ABBILDUNG 1:**
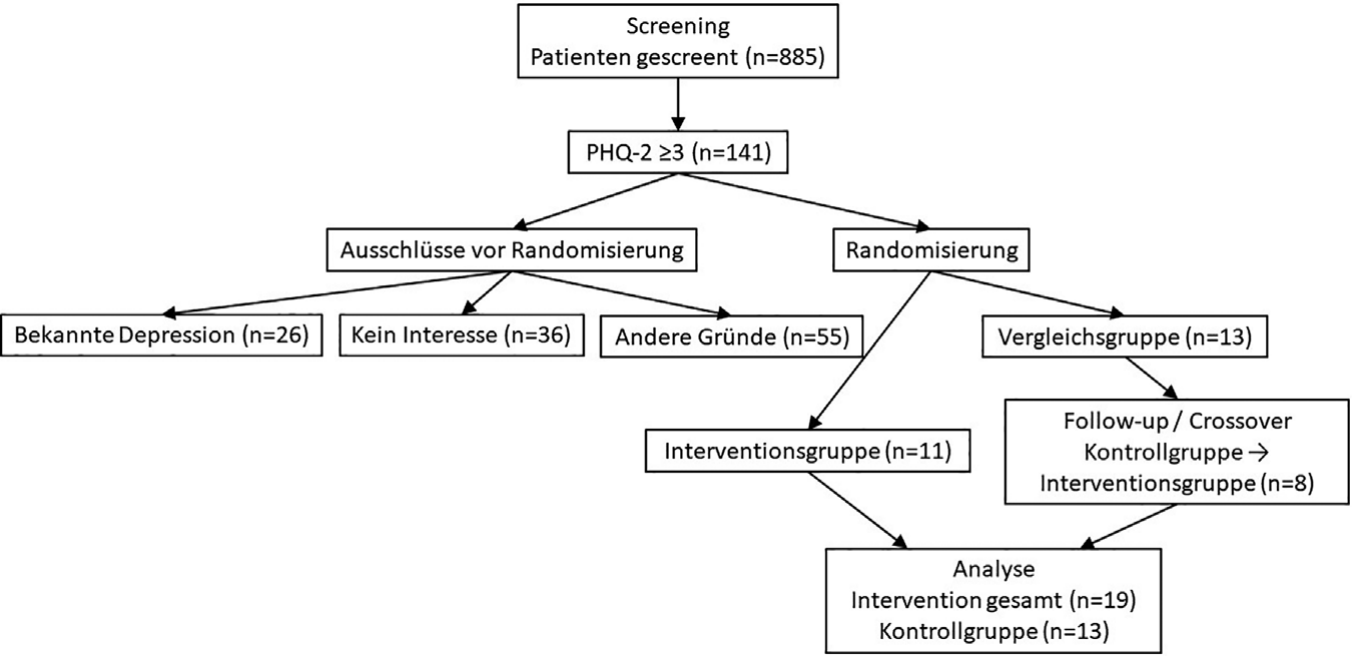
CONSORT‐Flussdiagramm zur Darstellung des Screenings der Teilnehmenden, der Ausschlüsse, der Randomisierung, der Nachverfolgung und der Analyse.

**TABELLE 2:** Stichprobenmerkmale.

Patienten	Gesamt n = 24
Alter in Jahren (MW ± SD)	47,8 ± 13,3
Geschlecht; weiblich n	13
Topische Therapie; n	4
Systemisch orale Therapie; n	5
Systemisch s.c. Therapie; n	15
PHQ‐9 Baseline (MW ± SD)	10,3 ± 4,47
*Psoriasis; n*	*18*
*Interventionsgruppe*	
PASI Baseline (MW ± SD)	2,8 ± 1,5
PASI Studienende (MW ± SD)	2,7 ± 1,9
*Kontrollgruppe*	
PASI Baseline (MW ± SD)	5,7 ± 4,3
PASI Studienende (MW± SD)	3,5 ± 3,4
*Atopische Dermatitis; n*	*6*
*Interventionsgruppe*	
EASI Baseline (MW± SD)	7,8 ± 9,1
EASI Studienende (MW± SD)	8,4 ± 10,0
*Kontrollgruppe*	
EASI Baseline (MW± SD)	8,0 ± 7,2
EASI Studienende (MW± SD)	3,4 ± 0,4

*Abk*.: EASI, Eczema Area and Severity Index; MW, Mittelwert; PASI, Psoriasis Area and Severity Index; PHQ‐9, Patient Health Questionnaire‐9; s.c., subkutan; SD, Standardabweichung

Die Verbesserung des PHQ‐2 war das Hauptzielkriterium der vorliegenden Studie. Zusätzlich wurde die Wirkung der Ultra‐ISTST‐Intervention anhand der psychometrischen Fragebögen DLQI, PHQ‐9, WHO‐5 und BDI‐II analysiert. Die Interventionsgruppe zeigte nach der Intervention (V4) eine statistisch signifikante Verbesserung des PHQ‐2 (p = 0,002) und des DLQI (p = 0,014) im Vergleich zum Ausgangswert (V1) (Abbildung 2), während in der Kontrollgruppe nur der PHQ‐2‐Wert signifikant beeinflusst war (p = 0,034). Um festzustellen, ob sich die Verbesserung der Lebensqualität nach der Intervention fortsetzte, wurden die Patienten 3 Monate nach Ende der Intervention erneut befragt (V5). Die Analyse aller ausgewerteten psychologischen Fragebogenwerte zeigte, dass die signifikante Verbesserung am Ende der Intervention im PHQ‐2 (p = 0,001) auch 3 Monate nach der Intervention anhielt und im Fall des WHO‐5 (p = 0,015) sich nach 3 Monaten nur im Vergleich zum Ausgangswert signifikant verbessert hatte (Abbildung 2).

**ABBILDUNG 2:**
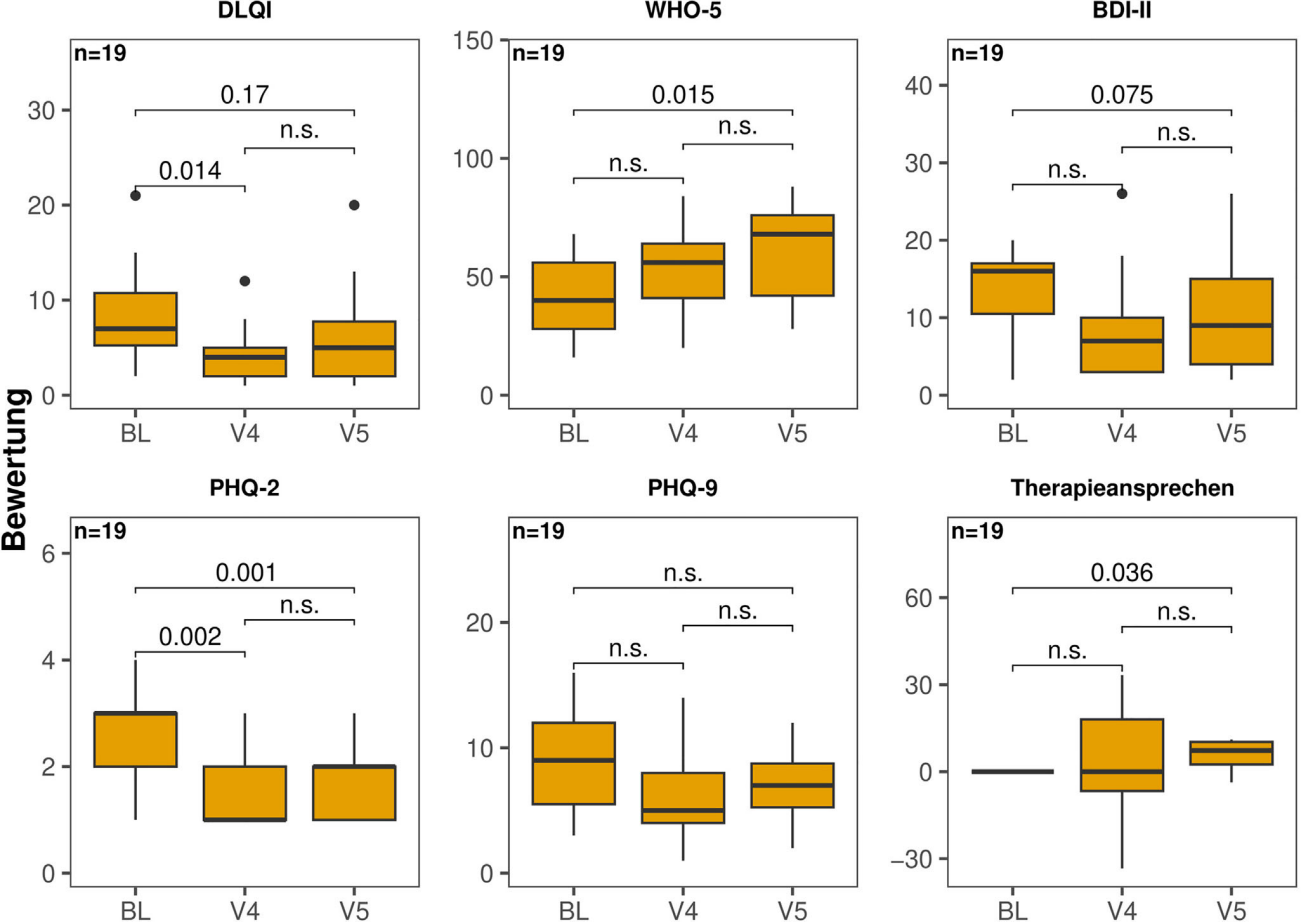
Ergebnisse der Fragebögen der Interventionsgruppe (n = 19) im Verlauf der Intervention. Die Fragebögen DLQI, WHO‐5, BDI‐II, PHQ‐2 und PHQ‐9 wurden mit den jeweiligen Instrumenten ausgewertet. Dermatologische Rückmeldungen wurden bei V4 (6 Wochen nach Studienbeginn) und V5 (12 Wochen nach Studienbeginn) als prozentuale Veränderung gegenüber dem Ausgangswert bestimmt. Die p‐Werte geben die Ergebnisse des Wilcoxon‐Rangsummentests zwischen den einzelnen Zeitpunkten an. n = Anzahl der Teilnehmer.

Der direkte Vergleich der Interventionsgruppe mit der Kontrollgruppe nach der Intervention (V4) ergab signifikant bessere Werte im PHQ‐2 (p = 0,005), DLQI (p = 0,003), BDI‐II (p = 0,001) und PHQ‐9 (p = 0,020) in der Interventionsgruppe im Vergleich zur Kontrollgruppe (Abbildung 3). Um festzustellen, ob die verbesserte Lebensqualität mit einer Verbesserung des klinischen Schweregrads der Erkrankung einherging, wurde die Verbesserung der klinischen Symptome anhand der prozentualen Veränderung des PASI (bei Psoriasis‐Patienten) oder des EASI (bei AD‐Patienten) analysiert. Es zeigte sich, dass sich die klinischen Symptome sowohl in der Interventions‐ als auch in der Kontrollgruppe im Vergleich zum Ausgangswert verbesserten, jedoch war die Verbesserung in der Interventionsgruppe im Vergleich zur Kontrollgruppe bei Besuch 4 signifikant größer (p = 0,039) (Abbildung 3).

**ABBILDUNG 3:**
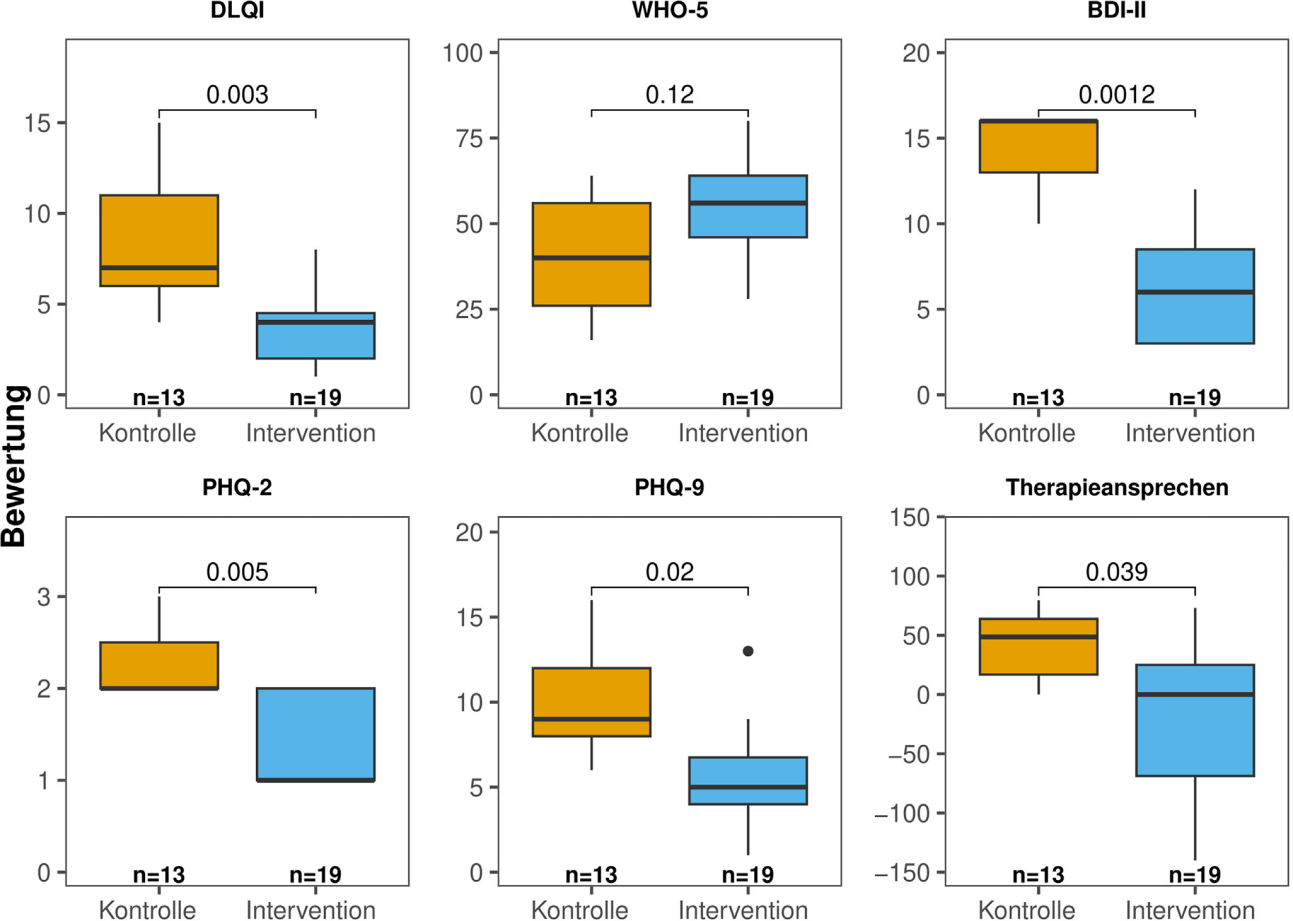
Ergebnisse der psychologischen Fragebögen und der klinischen Symptomschwere zum Zeitpunkt V4 im Vergleich zwischen Kontrollgruppe (n = 13) und Interventionsgruppe (n = 19). Die Fragebögen DLQI, WHO‐5, BDI‐II, PHQ‐2 und PHQ‐9 wurden mit den jeweiligen Instrumenten ausgewertet. Die dermatologische Rückmeldung wurde bei V4 als prozentuale Veränderung gegenüber dem Ausgangswert bestimmt. Als klinische Schweregradmessung wurden bei atopischer Dermatitis der Eczema Area and Severity Index (EASI) und bei Psoriasis der Psoriasis Area and Severity Index (PASI) verwendet. Die p‐Werte geben die Ergebnisse des Wilcoxon‐Rangsummentests zwischen den Gruppen an. n = Anzahl der Teilnehmenden.

Lineare gemischte Modelle (LMM) wurden verwendet, um die abhängigen Zusammenhänge der Ergebnisvariablen der Fragebögen mit der Durchführung der Intervention zu bestimmen. Die statistische Analyse der LMM zeigte, dass die psychotherapeutische Intervention einen signifikanten Einfluss auf den PHQ‐2 sowie auf den DLQI, den WHO‐5, den BDI‐II und den PHQ‐9 hatte (Abbildung 4). Im nächsten Schritt wurden diese Modelle um das klinische Ansprechen (prozentuale Veränderung des EASI/PASI) als abhängige Variable erweitert. Es zeigte sich, dass die beobachtete Veränderung der somatischen Befunde während der Intervention selbst keinen signifikanten Einfluss auf die Verbesserung der psychischen Gesundheit und Lebensqualität hatte (Abbildung 5). Somit konnte gezeigt werden, dass die Verbesserung der depressiven Symptome, der Lebensqualität und des psychischen Wohlbefindens durch die Ultra‐ISTST unabhängig von einer Verbesserung des klinischen Bildes war.

**ABBILDUNG 4:**
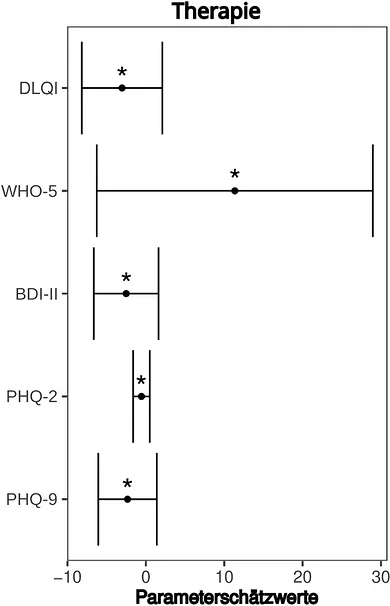
Effektstärken der Therapie auf die Ergebnisse der V4‐Fragebögen. Die Effektstärken wurden auf Basis linearer gemischter Modelle bestimmt. Die Signifikanzkennzeichnung zeigt den Vergleich mittels Varianzanalyse (ANOVA) des Therapiemodells (score ∼ group × visit + [1|patient]) gegenüber dem Nullmodell (score ∼ [1|patient]). *p < 0,05.

**ABBILDUNG 5:**
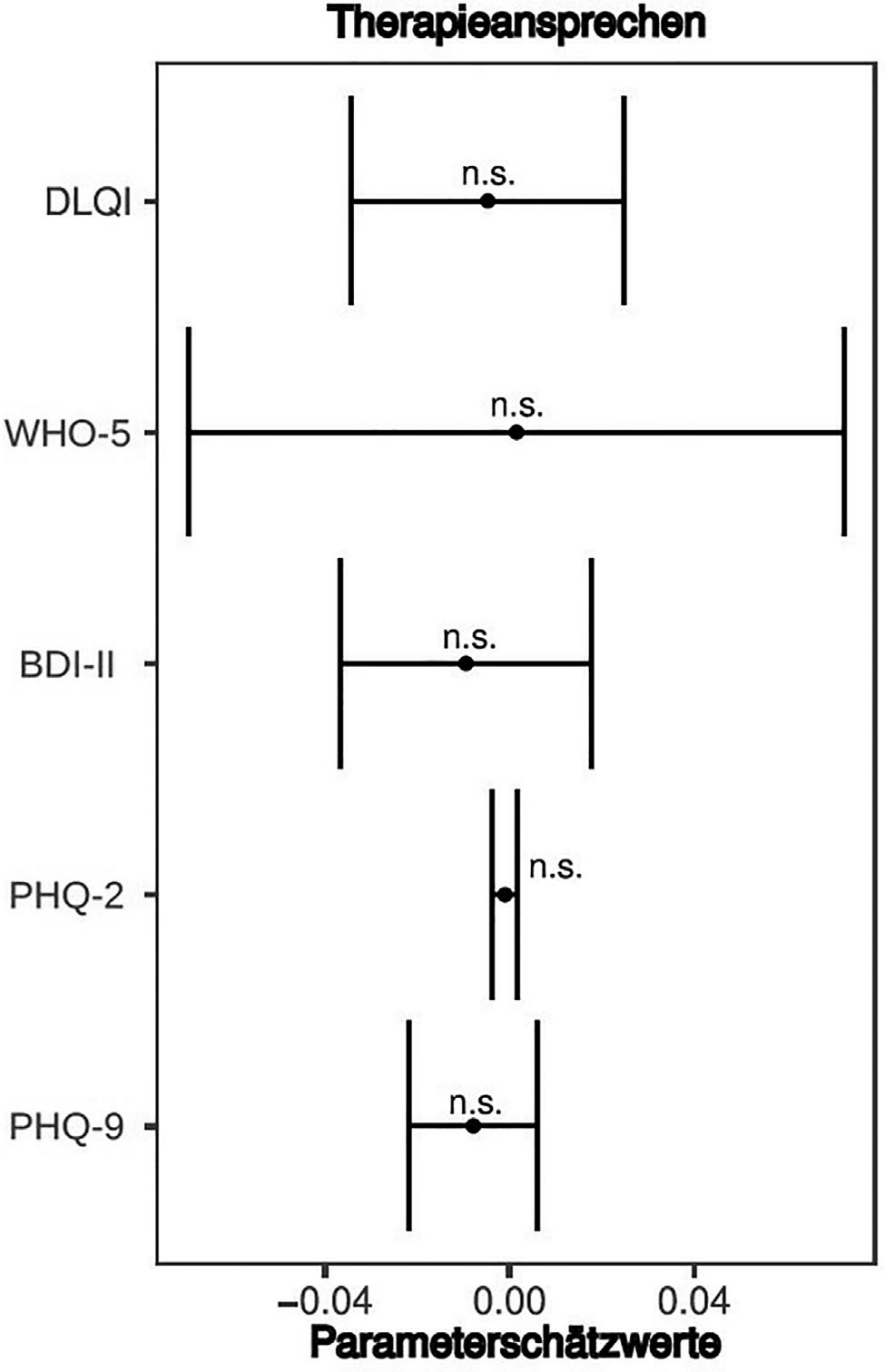
Effektstärken der klinischen Rückmeldung auf die Ergebnisse der V4‐Fragebögen. Die Effektstärken wurden auf Basis linearer gemischter Modelle bestimmt. Die Signifikanzkennzeichnung zeigt den Vergleich mittels Varianzanalyse (ANOVA) des Response‐Modells (score ∼ Gruppenzugehörigkeit × visit + klinische Rückmeldung + [1|patient]) gegenüber dem Behandlungsmodell (score ∼ Gruppenzugehörigkeit × visit). *p < 0,05.

Um die Wirksamkeit des Ultra‐ISTST zusätzlich zu beurteilen, wurde die Qualität der Intervention nach Beendigung der letzten Einzelsitzung anhand eines speziell entwickelten und standardisierten Bewertungsbogens überprüft und deskriptiv ausgewertet (Abbildung 6). Aus den Freitexten konnten insgesamt 46 Aussagen kategorisiert werden, die vier Hauptkategorien und entsprechenden Subkategorien zugeordnet werden konnten. Vierundzwanzig Aussagen bezogen sich auf die Hauptkategorie „Alltagstauglichkeit der Sitzungen”. Die Teilnehmer fokussierten auf die Bewusstmachung der individuellen Problematik, die Konkretisierung und die gemeinsame Lösungsfindung. Ein Teilnehmer schrieb: „Ich habe mit dem individuellen Fallkonzept meine eigenen Stressfaktoren herauszuarbeitet, damit ich in Zukunft besser mit ihnen umgehen kann […] und […] beachte”. Insgesamt wurden neun Aussagen unter der Hauptkategorie „Therapeuten‐Patienten‐Beziehung“ generiert, von denen sechs Aussagen der Subkategorie „sicher und geborgen gefühlt“ zugeordnet werden konnten. Acht Aussagen wurden für die Hauptkategorie „Erlernen spezifischer Techniken“ generiert. Die Patienten betonten die typischen Techniken aus der schematherapeutischen Arbeit, wie Stuhldialog und Imagination. Zudem wurden fünf Aussagen von den Teilnehmenden als selbstwertstärkend beschrieben, wie zum Beispiel die Aussage: „[…] mir wurden meine Stärken gut ins Gedächtnis gerufen.“ (Tabelle 3).

**ABBILDUNG 6:**
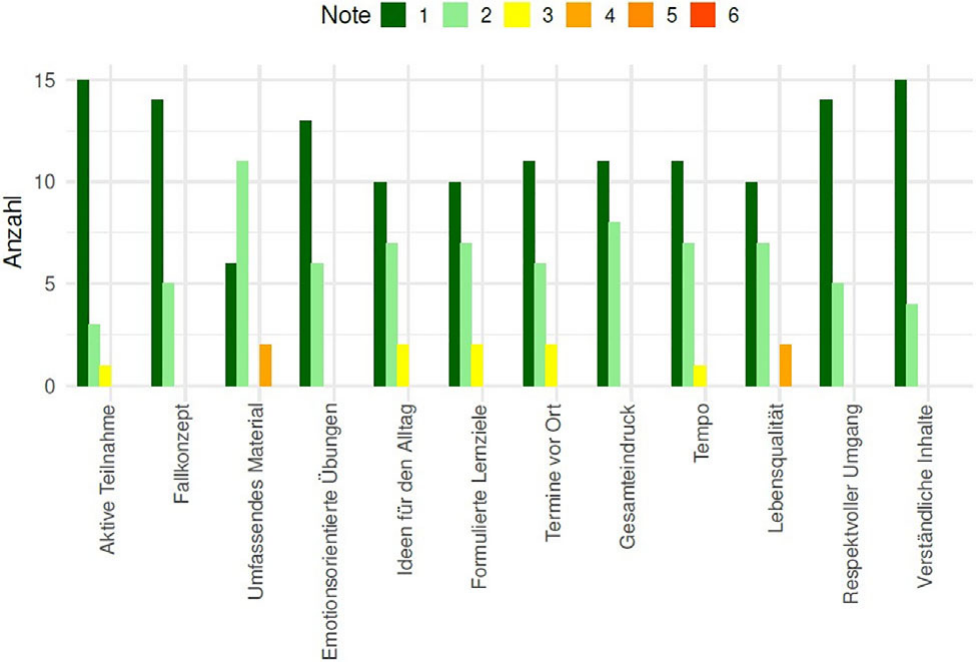
Bewertung des Moduls im deutschen Schulnotensystem aus Patientensicht. Die Teilnehmenden bewerteten das Modul nach dem Schulnotensystem (1 = sehr gut, 2 = gut, 3 = befriedigend, 4 = ausreichend, 5 = mangelhaft, 6 = ungenügend).

**TABELLE 3:** Verteilung der Aussagen im Kategoriensystem.

Hauptkategorie	Subkategorie	Aussagen (n)	Aussagen (%)
Alltagstauglichkeit der Sitzungen	Umsetzung der Inhalte im Alltag	4	16,7
	Bewusstmachung individueller Problematik	9	37,5
	Konkretisierung und Lösungsfindung	7	29,2
	Generell sinnvolle Themenauswahl	4	16,7
	*Gesamt*	24	100
Selbstwertstärkung	Eigene Stärken erkannt durch Reflektion	5	100
Erlernen von konkreten Techniken	Atemübung	1	12,5
	Stressreduktionstraining	1	12,5
	Stuhldialog	3	37,5
	Imagination	2	25,0
	Entspannungsübung	1	12,5
	*Gesamt*	8	100
Therapeuten‐Patienten‐Beziehung	Akzeptiert und wertgeschätzt gefühlt	1	11,1
	Sicher und geborgen gefühlt	6	66,7
	Möglichkeit sich zu öffnen und auszusprechen	2	22,2
	*Gesamt*	9	100

Während des Untersuchungszeitraums wurden in keiner der beiden Gruppen unerwünschte Nebenwirkungen beobachtet.

## DISKUSSION

Es wurden verschiedene psychotherapeutische Interventionsformate für Patienten mit chronischen Hauterkrankungen beschrieben, die von Trainingsprogrammen, psychodynamischen Therapien, kognitiven Verhaltenstherapien, achtsamkeitsbasierten Interventionen, Stressbewältigungs‐ und Entspannungstechniken bis hin zu systemischen Ansätzen und Langzeitpsychotherapien in Leitlinienverfahren reichen.^19^, ^35^, ^36^, ^37^ In der vorliegenden Arbeit wird die von uns entwickelte ULTRA‐ISTST vorgestellt – ein schematherapeutischer Ansatz, der darauf abzielt, psychisch Belasteten ohne manifeste Depression im dermatologischen Kontext ein niedrigschwelliges Behandlungsangebot bereitzustellen. Die Kurzzeitintervention, bestehend aus drei manualbasierten 50‐minütigen Therapiesitzungen, zeigte im Vergleich zu einer Kontrollgruppe eine signifikante Wirksamkeit bei der Verbesserung depressiver Symptome und der verminderten Lebensqualität.

Für eine umfassende Versorgung von Patienten mit Hautkrankheiten ist ein kostengünstiges Screening auf psychische Symptome im Rahmen der ambulanten somatischen Versorgung wünschenswert. Gerdes et al. konnten zeigen, dass die Whooley‐Fragen eine einfach anzuwendende Methode zum Screening auf Depressionen bei Psoriasis sind.^18^ Darüber hinaus konnten Köster et al. den frei verfügbaren PHQ‐4 zur Früherkennung psychischer Erkrankungen im stationären Kontext in die elektronische Patientenakte implementieren und so eine zeitnahe psychosomatische Beratung für die Betroffenen ermöglichen.^38^ Auf Grundlage dieser Ergebnisse haben wir in unserer Pilotstudie den frei verfügbaren PHQ‐2‐Fragebogen eingesetzt, um Betroffene zu identifizieren. Wir konnten erneut zeigen, dass es möglich ist, psychisch belastete Patienten im ambulanten Kontext zu erkennen. Mit Hilfe der Intervention konnten wir diesen Patienten unmittelbar nach dem Screening im Rahmen der dermatologischen Beratung eine niedrigschwellige Behandlung anbieten, von der ein beträchtlicher Teil erheblich profitieren konnte.

Psychosoziale Faktoren und Erkrankungen stellen eine besondere Herausforderung dar. Die Überweisung von psychisch belasteten Patienten in die ambulante Weiterbehandlung ist für behandelnde Dermatologen aufgrund fehlender interdisziplinärer Systeme in manchen Fällen oft schwierig.^39^ Die Integration von somatischer und psychotherapeutischer Behandlung ist von aktueller Bedeutung und spiegelt sich in der Umsetzung der Psychodermatologie wider. Bei chronisch‐entzündlichen Erkrankungen wurde ein neuroimmunologischer Zusammenhang zwischen somatischer Entzündung und psychologischer Komorbidität festgestellt.^40^ Daher wird heute ein multidisziplinärer Ansatz zur Behandlung von Patienten mit chronisch entzündlichen Hauterkrankungen bevorzugt.^41^

Kurzfristige Interventionen, insbesondere bei Patienten mit milden Symptomen ohne manifeste psychische Erkrankung, können eine Versorgungslücke zwischen ambulantem Bedarf und der leitliniengerechten Standardpsychotherapie schließen. Die Ultra‐ISTST‐Pilotstudie zeigte nach drei Behandlungsterminen eine signifikante Verbesserung der Lebensqualität und eine Verringerung der depressiven Symptome, unabhängig von einer Verbesserung der Hautbefunde im Rahmen der Standardtherapie. Das hier vorgestellte Konzept sollte jedoch nicht als Ersatz für etablierte Therapieformate angesehen werden, sondern als niedrigschwelliges Unterstützungsprogramm, das in die primäre dermatologische Versorgung integriert werden kann. Die psychosomatische Grundversorgung kann hier eine entscheidende Rolle spielen.^42^ Die psychosomatische Grundversorgung ist kein Pflichtbestandteil der fachärztlichen Weiterbildung zum Dermatologen, kann jedoch optional erworben werden, wenn Leistungen der psychosomatischen Grundversorgung abgerechnet werden sollen.^43^ Die in unserer Studie eingesetzte manualbasierte und zeitlich begrenzte Intervention könnte als Grundlage für interessierte Dermatologen dienen, die in ihrer täglichen Praxis eine qualitätsgesicherte Kurzzeitintervention anbieten möchten. Eine besondere Stärke unserer Ultra‐ISTST ist die gemessene Nachhaltigkeit der Intervention. Die Teilnehmer in der Interventionsgruppe zeigten auch 3 Monate nach Ende der Intervention eine konstante Verbesserung ihrer depressiven Symptome und ihrer Lebensqualität. Die Patienten berichteten, insbesondere davon profitiert zu haben, in einem sicheren therapeutischen Rahmen ihre individuellen Belastungsfaktoren zu reflektieren und spezifische Techniken kennenzulernen. Dieser ganzheitliche Ansatz verhalf den Betroffenen zu einem besseren Verständnis der erlebten Stigmatisierung.^44^ Ausgehend von der Annahme, dass Patienten mit Hauterkrankungen unter Stress zu dysfunktionalen Bewältigungsstrategien neigen, kann ein vertieftes Verständnis ihrer individuellen Fallkonzeption dazu beitragen, die Motivation zu fördern, adaptive und konkrete alternative Bewältigungsansätze zu entwickeln und in den Alltag zu integrieren.^45^

Eine wichtige Erkenntnis dieser Studie war, dass die beobachteten Verbesserungen der psychischen Gesundheit und Lebensqualität weitgehend unabhängig von Veränderungen des Schweregrads der Hauterkrankung waren. Während des Studienzeitraums wurden in keiner der beiden Gruppen unerwünschte Nebenwirkungen beobachtet. Dies deutet darauf hin, dass Ultra‐ISTST spezifische psychosoziale Bedürfnisse adressieren kann, die nicht direkt mit dem klinischen Hautzustand zusammenhängen.

Diese Pilotstudie weist mehrere Limitationen auf. Erstens wurden bei den wichtigsten Analysen zwischen den Gruppen alle Teilnehmer, die die Intervention erhielten, unabhängig von ihrer ursprünglichen Randomisierung zusammengefasst, um die Stichprobengröße für die Schätzung der Effektgrößen zu maximieren. Dieser Ansatz ist zwar im Rahmen einer Pilotstudie angemessen, weicht jedoch von einem streng randomisierten Vergleich ab und kann aufgrund einer möglichen Auswahlverzerrung zu einer Überschätzung der Behandlungseffekte führen. Zweitens wurde sie in einer spezialisierten Ambulanz durchgeführt, was die Übertragbarkeit der Ergebnisse auf andere Versorgungssettings einschränken könnte. Drittens war die Studie nicht verblindet und umfasste keine aktive Kontrollintervention, sodass unspezifische Behandlungseffekte nicht ausgeschlossen werden können. Viertens war die Stichprobengröße klein und wurde pragmatisch ohne formale Leistungsberechnung ausgewählt, was die statistische Aussagekraft zur Erkennung kleinerer Effekte einschränkt. Fünftens wurden zwar keine interventionsbedingten unerwünschten Ereignisse gemeldet, aber unerwünschte Ereignisse wurden nicht systematisch überwacht, obwohl der allgemeine Gesundheitszustand der Teilnehmer bei jedem Besuch überprüft wurde.

Die Patientenpopulation stammte aus einem tertiären Dermatologiezentrum in Deutschland, und die meisten Teilnehmer erhielten bereits eine leitlinienbasierte dermatologische Versorgung. Die manualisierte Natur der Intervention ermöglicht zwar eine Anpassung an breitere Kontexte, ihre Durchführbarkeit in der Primärversorgung oder in Versorgungsbereichen ohne unmittelbaren Zugang zu psychotherapeutischer Expertise muss jedoch noch evaluiert werden.

Implikationen für die zukünftige Forschung

Als Pilotstudie bestand das Hauptziel darin, die Durchführbarkeit zu bewerten und Informationen für die Konzeption einer größeren, endgültigen randomisierten kontrollierten Studie zu liefern. Zukünftige Studien sollten ein multizentrisches Design in Betracht ziehen, eine aktive Kontrollgruppe einbeziehen, eine längerfristige Nachbeobachtung für beide Gruppen durchführen und potenzielle unerwünschte Ereignisse systematisch überwachen.

## DANKSAGUNG

Wir möchten uns bei allen Patienten des Zentrums für entzündliche Hauterkrankungen bedanken, die sich bereit erklärt haben, an der Studie teilzunehmen und damit deren Durchführung ermöglicht haben.

Open access Veröffentlichung ermöglicht und organisiert durch Projekt DEAL.

## INTERESSENKONFLIKT

Keiner.
